# Different forms of nitrogen uptake in tobacco promoted by the arbuscular mycorrhizal fungi

**DOI:** 10.3389/fpls.2025.1600887

**Published:** 2025-06-30

**Authors:** Yanlan Xie, Xiaohui Song, Yingang Lu, Xianfeng Hu, Shouhui Pan, Wei Xu, Yuan Xue

**Affiliations:** ^1^ College of Agriculture, Anshun University, Anshun, Guizhou, China; ^2^ College of Agriculture, Guizhou University, Guiyang, Guizhou, China; ^3^ Guizhou Province Tobacco Company Anshun Company, Anshun, Guizhou, China

**Keywords:** arbuscular mycorrhizal fungi, nitrogen, tobacco, absorbance mismatch, 15N

## Abstract

Persistent limitations in nitrogen (N) assimilation efficiency have emerged as a critical constraint in advancing the phytochemical quality of cultivated tobacco (*Nicotiana tabacum* L.). Arbuscular mycorrhizal fungi (AMF), forming obligate symbiotic associations with over 80% of terrestrial vascular plant species, significantly enhance host plant performance through improved rhizospheric nutrient mobilization. This mutualistic relationship facilitates enhanced acquisition of both macronutrients (particularly phosphorus and N) and water, thereby substantially decreasing agricultural dependence on synthetic fertilizer inputs. Building upon these premises, the present study was carried out to investigate the effects of different forms of nitrogen on the infestation rate and biomass of tobacco plants after inoculation with AMF, as well as the differences in the uptake of different forms of nitrogen by tobacco plants mediated by AMF, using the isotope ^15^N labelling method. The study revealed significant variations in the uptake of various nitrogen forms by AMF. Under mixed nitrogen source conditions, (NH_4_)_2_SO_4_, KNO_3_, and glutamine (Glu) constituted 48.61%, 36.10%, and 15.29% of total nitrogen uptake, respectively. Notably, AMF exhibited a preferential uptake hierarchy for NH_4_^+^, demonstrating 1.35-fold and 2.94-fold higher absorption rates compared to NO_3_^-^ and Glu. Furthermore, ^15^N isotopic tracing analysis confirmed active Glu assimilation by AMF, as evidenced by significantly elevated ^15^N-Glu uptake in labeled treatments relative to non-labeled controls. These findings collectively suggest that AMF symbiosis modifies tobacco plants’ nutritional preferences among distinct nitrogen forms. This study establishes a theoretical foundation for optimizing nitrogen utilization efficiency and enhancing agronomic productivity in tobacco cultivation systems.

## 1 Introduction

Nitrogen (N) is one of the most important mineral nutrients essential for plant growth and development. In most terrestrial ecosystems, N serves as a constraint on net primary production (Martinez-Feria et al., 2018). Non-leguminous plants are usually incapable of utilizing N_2_ for their own growth. Consequently, the external application of chemical N fertilizers becomes a crucial method of artificially replenishing the soil nitrogen pool (Calabrese et al., 2017; Guzman-Bustamante et al., 2019).Nevertheless, the utilization of chemical N fertilizers by plants is typically minimal, accounting for only 35% of the total N input. The remainder is lost through various processes, including water pollution, rain-induced leaching and the emission of greenhouse gases nitrous oxide (N_2_O) (Martinez-Feria et al., 2018; Zeng et al., 2021). Therefore, enhancing nitrogen use efficiency and promoting sustainable agricultural practices remain critical challenges in modern agriculture.

Soil microorganisms have been demonstrated to exert a pivotal influence on the interaction between nutrient turnover and crop metabolism (Fang et al., 2025). Arbuscular mycorrhizal fungi (AMF), as a biofertilizer, underscoring its capacity to enhance soil fertility and plant growth and development, thereby reducing the reliance on chemical fertilizers and enhancing their utilization (Qian et al., 2024). It is widely recognized that AMF stimulates plants growth by facilitating the uptake of large amounts of essential nutrients while reducing sodium and chloride uptake (Begum et al., 2019). More than 80% of terrestrial flora, including flowering plants, bryophytes, and ferns, can form a mutually beneficial symbiotic relationship with AMF (Zhang et al., 2024). This interaction serves to enhance plant nutrient uptake and to maintain the carbon and nitrogen balance within the ecosystem (Li et al., 2019; Wang et al., 2022). Host plants provide AMF with a carbon source to meet their energy requirements for growth and build the carbon skeleton of fungal cells (Begum et al., 2019). Contrarily, AMF facilitate the growth of plants by providing access to essential mineral nutrients in the soil, as well as by mitigating biotic or abiotic stresses (Shi et al., 2020; Willing et al., 2024). Mycorrhizal plants utilize two different mechanisms for nutrient uptake: directly through plant roots and indirectly through the AMF hyphal network (Ward et al., 2023; Wang et al., 2024). Mycorrhizal plants use the mycorrhizal pathway to absorb nutrients such as nitrogen, phosphorus, potassium, copper and silicon (Senovilla et al., 2020; Begum et al., 2021a; Pu et al., 2022; Qiu et al., 2024). This fact was confirmed that approximately 20 to 75% of the total N uptake by AMF plants can be transferred to host plants via the mycorrhizal symbiosis pathway (Hashem et al., 2018; Wang et al., 2024).

In terrestrial ecosystems, when the hyphal network formed by AMF connects plants of the same or different species, the hyphal network effectively distributes the absorbed nutrients according to the specific nutrient requirements of each plant (Montesinos-Navarro et al., 2019; de Leon et al., 2024). For example, in the hyphal network between flax and sorghum, AMF absorbs minimal C nutrients from flax but provides up to 94% of N and P to flax through the hyphae, which stimulates flax growth. Conversely, AMF obtains a substantial amount of C from sorghum but allocates a relatively small quantity of N and P to sorghum (Walder et al., 2015). Similarly, subterranean hyphal networks have been observed to regulate the redistribution of NH_4_^+^ and NO_3_^-^ among plants, including between nitrogen-fixing and non-nitrogen-fixing plants, and between herbaceous and woody plants (Qian et al., 2024). Most mycorrhizal plants exhibit a higher proportion of assimilated NH_4_^+^ to total nitrogen compared to non-mycorrhizal plants, due to the direct absorption of NH_4_^+^ via the GS/GOGAT pathway (Wang et al., 2022). Some scientists have reported that ammonium and phosphate transporters are markedly present in the plasma membrane of root cells in mycorrhizalized plants (Cheng et al., 2022; Han et al., 2023). The existence of these transport proteins induces mycorrhizal plants to transport ammonium nitrogen 10 times faster than nitrate nitrogen (Wang et al., 2022). It can be reasonably deduced that ammonium nitrogen plays a pivotal role in plant symbiosis.

Tobacco (*Nicotiana tabacum* L.), serving as a key economic driver in economically underdeveloped regions of central and western China, holds dual agricultural significance (Begum et al., 2021a, 2021b). As the first genetically modified plant model, it facilitates functional validation of biotic/abiotic stress-resistance genes (Liu et al., 2020). Additionally, tobacco has also been selected for intercropping, e.g., with insectary floral plants to reduce nitrogen fertilizer inputs and improve chemical properties of flue-cured tobacco (Zhong et al., 2024). Tobacco acreage decreased by 24.36% and nitrogen fertilizer application decreased by 25.83% in 2023 compared to 2000, but production per unit area increased by only 17.13%, according to the National Statistical Office (NSO). This suggests that the uptake and utilization of nitrogen fertilizer in tobacco needs to be further improved.

Nitrogen sufficiency directly affects the chemical composition of tobacco and thus its yield (Liu et al., 2025; Tian et al., 2025). AMF has been demonstrated to facilitate tobacco plant growth by promoting water, mineral and nutrient uptake (de Leon et al., 2024; Zeng et al., 2025). Apart from this, root growth and nicotine synthesis of tobacco as affected by exposure to different nitrogen forms (Li et al., 2025). Consequently, the present study was undertaken to examine the preference of AMF strain for different forms of nitrogen uptake in tobacco. To this end, a ^15^N isotope tracer method was employed, utilizing AMF as the inoculum. The aim was to offer theoretical and practical guidance for the enhancement of tobacco yield quality and the optimization of fertilizer formulations.

## 2 Materials and methods

### 2.1 Plant material and growth conditions

The tobacco variety employed was China Tobacco 100, and the coated seeds were cultivated to reach the five-leaf and one-heart growth stages using the floating seedling method. Subsequently, uniform seedlings were selected and cultivated in columnar pots (π*72*14 cm) containing 2 kg of soil. The test soil was collected from 0~20 cm surface soil (106°44′53″N, 26°27′21″E) at the mountain behind the West Campus of Guizhou University. The test soils were subjected to autoclaving in order to eradicate the indigenous AMF population. The soil sample used for the test was a yellow soil with the following basic physical and chemical properties: water content 27.1%, pH 5.85, organic matter 21.6 g/kg, total nitrogen 1.08 g/kg, total phosphorus 0.75 g/kg, total potassium 11.6 g/kg, alkaline dissolved nitrogen 53.7 mg/kg, available phosphorus 12.9 mg/kg, available potassium 83.25 mg/kg. This experiment was conducted in a light culture room (light intensity: 3700lx) in which the room temperature was set at 25°C in daytime and 20°C at night.

**Figure 1:**
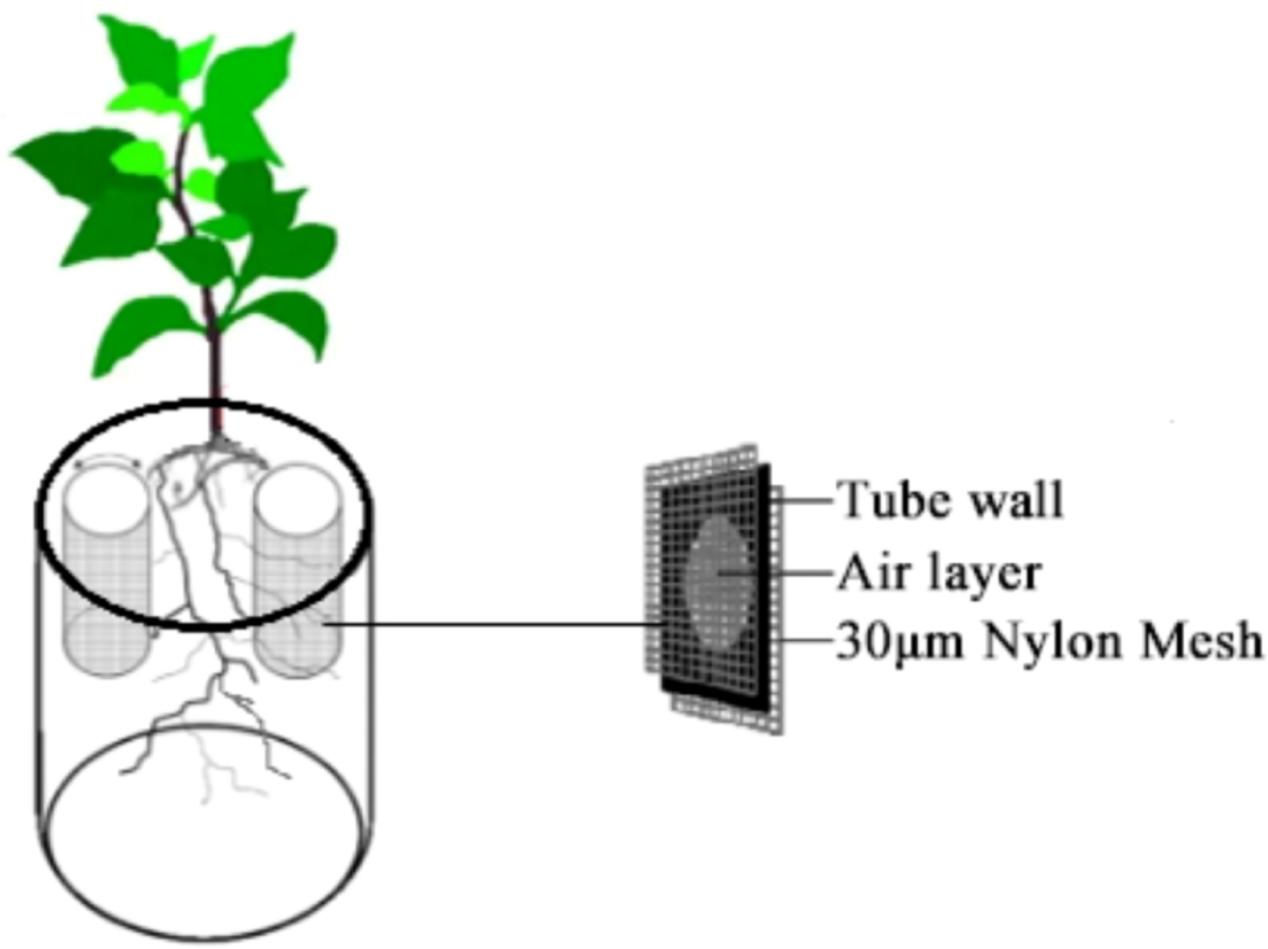
Schematic diagram of the test setup.

A Claroideoglomus etunicatum (Ce) strain was employed as a qualified inoculant for the 131d expansion of white clover, with a spore concentration of >50 spores/g. This strain was selected based on its high affinity for the Chinese Tobacco 100 variety, which is more favorable for tobacco dry matter accumulation, nutrient uptake, and up-regulation of nitrogen metabolism gene expression (Li et al., 2021). A one-way, three-level pot experiment was conducted to investigate the influence of different nitrogen forms (ammonium sulphate: (NH_4_)_2_SO_4_, potassium nitrate: KNO_3_ and glutamate: Glu) on the growth of plants. The experiment was conducted with an application rate of 30 g/kg of Ce to the soil. Polyvinyl chloride (PVC) tubes (π*22*10 cm) with perforations in the walls were placed within the pots on either side of the tobacco plants. The tubes were affixed with 30 μm nylon mesh on both the interior and exterior surfaces of the tube walls. The schematic diagram of the experimental setup is shown in **Figure 1**.

The nitrogen sources subjected to analysis were analytically pure (NH_4_)_2_SO_4_, KNO_3_ and Glu, along with the ^15^N markers of all three. The nitrogen nutrient solutions were formulated as follows: N0 treatment: (NH_4_)_2_SO_4_, KNO_3_ and Glu; N1treatment: (^15^NH_4_)_2_SO_4_, KNO_3_ and Glu; N2 treatment: (NH_4_)_2_SO_4_, K^15^NO_3_ and Glu; N3 treatment: (NH_4_)_2_SO_4_, KNO_3_ and Glu-^15^N. The nitrogen content was 0.33 mol N/L in each treatment. A total of 16 treatments were applied in this experiment with four replicates per treatment. The pot experiment was supplemented with distilled water at regular intervals, and the mycelium chamber was injected with Hoagland nitrogen-free nutrient solution on a weekly basis. Following a period of 60 days, 20 mL of the aforementioned nitrogen nutrient solution was injected into the mycelium chamber in accordance with the treatments employed in the experiment. The nutrient solution was composed of CaNO_3_·4H_2_O 945 mg/L, KNO_3_ 506 mg/L, NH_4_NO_3_ 80 mg/L, KH_2_PO_4_ 136 mg/L, MgSO_4_ 493 mg/L, KI 0.83 mg/L, H_3_BO_3_ 6.2 mg/L, MnSO_4_ 22.3 mg/L, ZnSO_4_ 8.6 mg/L, Na_2_MoO_4_ 0.25 mg/L, CuSO_4_ 0.025 mg/L, CoCl_2_ 0.025 mg/L, FeSO_4_·7H_2_O 5.56 g/L、EDTA-Na 7.46 g/L. Uniform healthy seedlings were selected and transplanted into pots (one plant per pot) on the day of fungicide application. The Ce fungicide was administered maintaining a 0.5 cm distance from seedling roots to prevent direct contact. The 1/2-strength Hoagland’s solution was applied to standardize nutrient availability, with soil moisture maintained at 40%. Post-transplantation care included weekly applications of 200 mL full-strength Hoagland’s solution starting at 7 days after transplanting, while adjusting soil moisture to 60% field capacity. Pots were arranged in a randomized complete block design with 40 cm × 40 cm spacing to minimize microenvironmental interference. To mitigate positional effects, all containers were rotated weekly and randomly repositioned within the growth chamber every fortnight. This randomization scheme effectively controlled potential light gradients and air circulation biases throughout the experiment. The entire plant was harvested after 7 days, and the plant was cultivated for a total of 67 days.

### 2.2 AMF colonization

The AMF colonization was determined using the microscopic observation method. The sections were prepared using the modified method (Zhang et al., 2015). Three tobacco plants were randomly selected from each treatment and the root system was completely extracted from the plant. The root tips were rinsed repeatedly with deionised water, and the young root tips of 1 cm of roots were cut for measuring the mycorrhizal infestation rate. The root tips were observed, recorded, photographed and measured using the taipan blue staining and square cross-hatching with a stereoscope (S9 DLeica, Germany). Here, the mycorrhizal infection rate (%) = (length of infected root segments/length of observed root segments) × 100%.

### 2.3 Determination of tobacco plant biomass

The cultivation process involved the random selection of each treatment of the growth of the tobacco plant, with the entire plant being removed from the culture pot. The height of the tobacco plant above ground was then measured using a tape measure, and the root system was carefully washed with deionized water to remove any debris. The root system of the tobacco plant was then cut off, and the maximum root length was measured with a straightedge. The aboveground and root systems of the tobacco plants were dried in an oven at 105°C for 30 min. Then further dried in an oven at 70°C until a constant weight is reached. Thereafter, the samples were weighed. Each treatment was conducted in triplicate.

### 2.4 Determination of nitrogen nutrient content of tobacco plants

The parts of the tobacco plant where the dry matter mass was determined were crushed and processed to determine total nitrogen. The total nitrogen content of the samples to be tested was determined using the Kjeldahl method (Quirino et al., 2023). Nitrogen uptake by the tobacco plant was calculated using the following formula: Nitrogen uptake by the tobacco plant (g/pot) = root nitrogen content of the tobacco plant × biomass of the tobacco plant.

### 2.5 Determination of labelled nitrogen ^15^N

An elemental analyzer-isotope ratio mass spectrometer (EA-IRMS, German) was employed for the determination of nitrogen derived from fertilizer (Ndff) in tobacco plants. Tobacco plant Ndff (%) = Tobacco plant ^15^N atomic weight/fertilizer ^15^N atomic weight ×100%.

### 2.6 Statistical analysis

The data were initially collated and analyzed using Microsoft Excel 2013. The assumptions of normality (verified by Shapiro-Wilk test) and homogeneity of variances (confirmed via Levene’s test) were satisfied prior to ANOVA implementation. All statistical analyses were performed using SPSS19.0 software (IBM SPSS Inc., Chicago, IL) (p < 0.05). Mean separations were determined using a *post hoc* Tukey’s multiple range test (p< 0.05). All data measurements were expressed as mean ± standard error (mean ± SE). All graphical representations were generated using Origin 8 software (OriginLab, Northampton, MA).

## 3 Results and analyses

### 3.1 Effect of different treatment conditions on AMF colonization on tobacco plants

**Figure 2:**
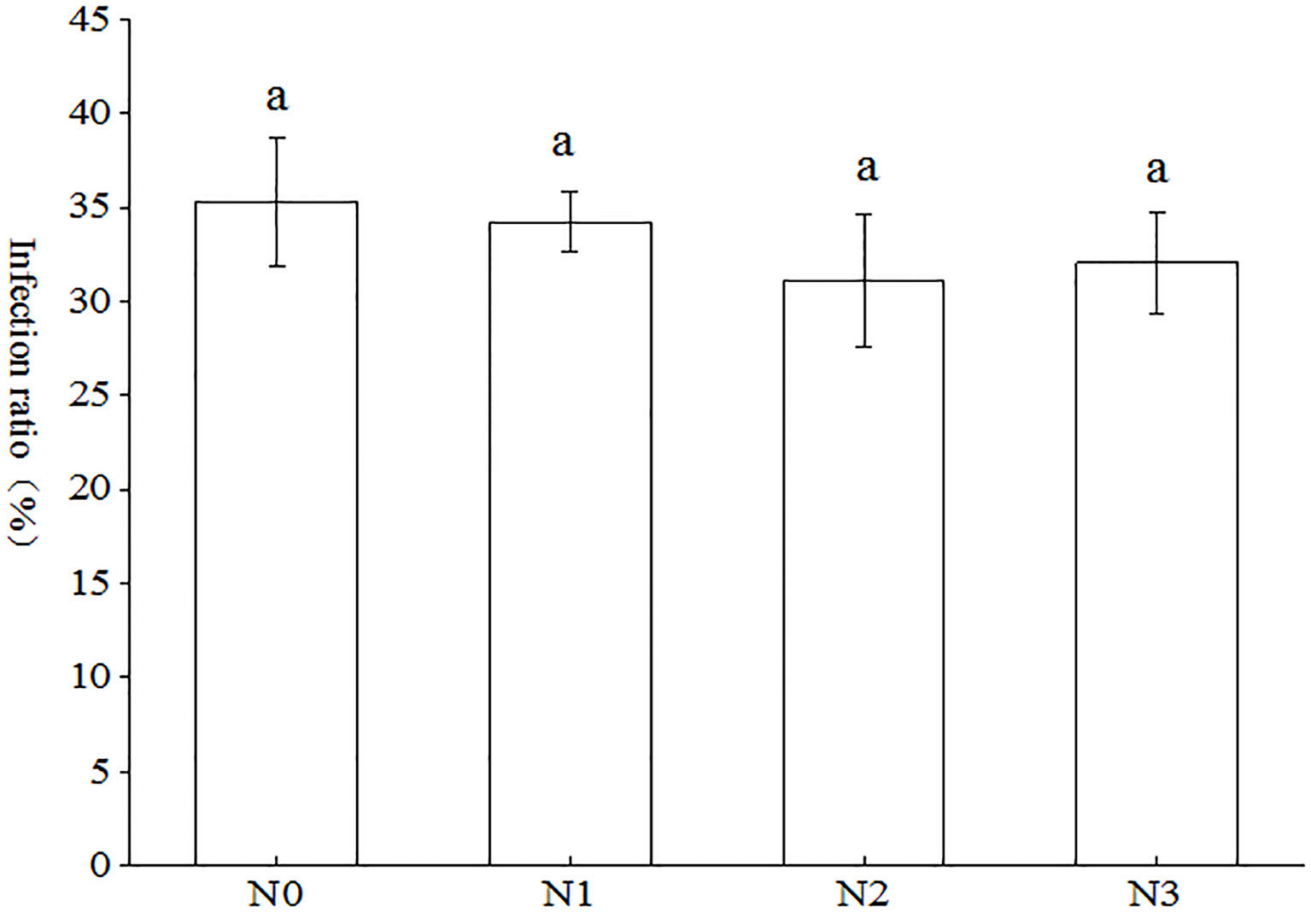
Effect of AMF on tobacco infection rate under different nitrogen forms. Different treatments are used with lower case letters (a, b, c) to indicate statistical significance (p<0.05).

As illustrated in **Figure 2**, the three distinct nitrogen tracer markers (NH_4_)_2_SO_4_, KNO_3_ and Glu were observed to facilitate the colonization of the root system of tobacco plants by AMF across all treatments, with infestation rates ranging from 31.13% to 35.29%. Notably, the N0 treatment exhibited the highest infestation rate, followed by N1 and N3, while N2 demonstrated the lowest infestation rate. In comparison to the N0 treatment, the N1, N2, and N3 treatments exhibited increases of 3.07%, 13.36%, and 10.11%, respectively. However, these differences did not reach the threshold for a statistically significant distinction between treatments (p < 0.05).

### 3.2 Effect of AMF inoculation on tobacco biomass accumulation

**Figure 3:**
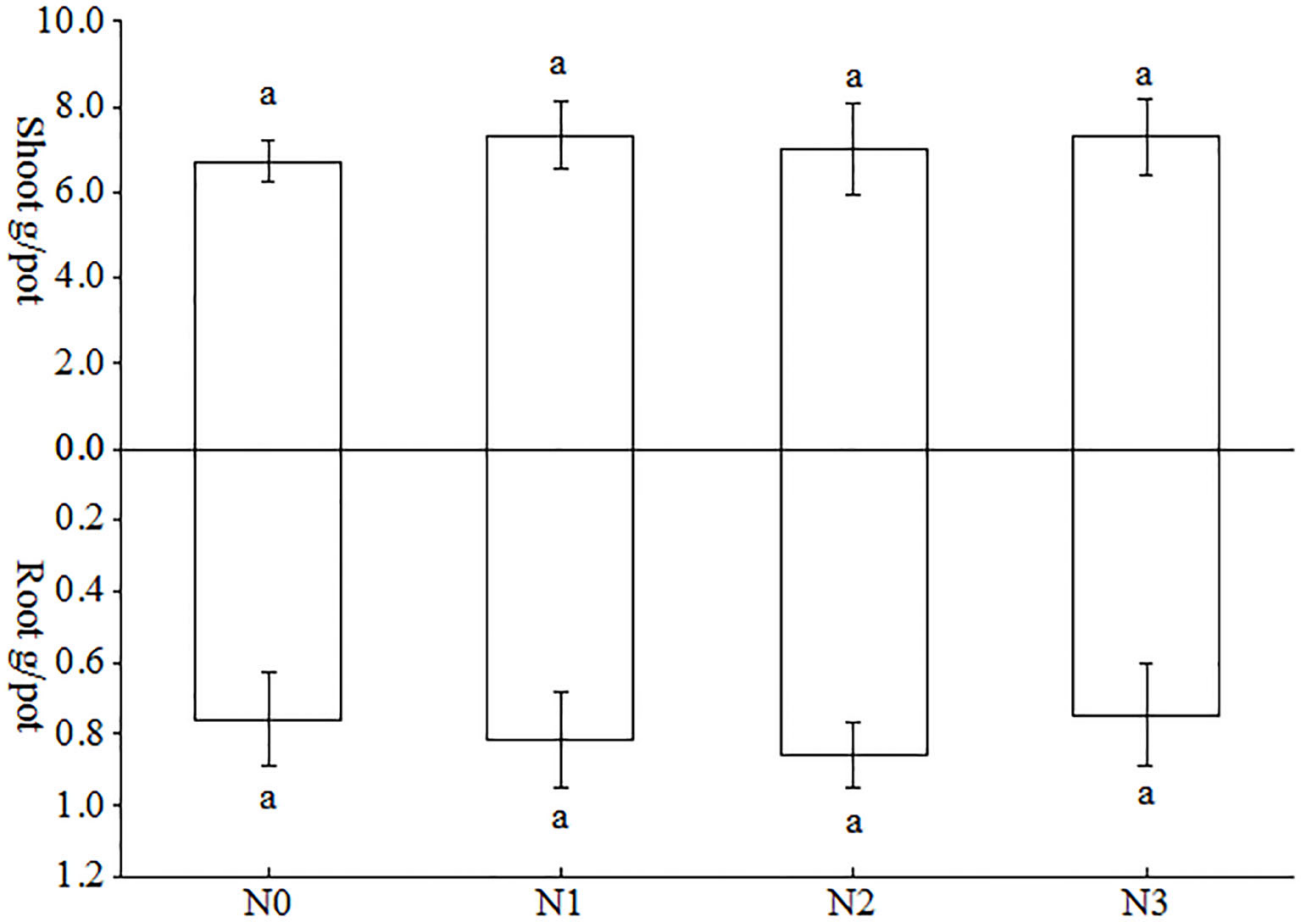
Effects of AMF on biomass accumulation in the above-ground and underground parts of tobacco plants under different forms of nitrogen. Different treatments are used with lower case letters (a, b, c) to indicate statistical significance (p<0.05).

As can be observed in **Figure 3**, the impact of varying nitrogen treatments on the accumulation of above- and below-ground biomass in tobacco plants was not uniform. In the aboveground part of the tobacco plant, the greatest increase in biomass was observed in N1 treatment, followed by N4 treatment, with increases of 9.24%, 4.56%, and 8.64%, 3.99%, compared with N0 and N3 treatments, respectively. However, there was no statistically significant difference between these two treatments. This may be attributed to the fact that the amount of nitrogen fertilizer and nitrogen source were the same for the different treatments in this study, only that the different nitrogen sources were isotopically labelled. Since the objective of this study was to investigate the uptake preference of tobacco inoculated with AMF for different forms of nitrogen. With regard to the subterranean portion of the tobacco plant, the N1 treatment exhibited a higher level, while the N3 treatment demonstrated a lower level. And neither of these levels reached the threshold for statistical significance.

### 3.3 Effect of AMF inoculation on the accumulation of nitrogen content in tobacco

**Figure 4:**
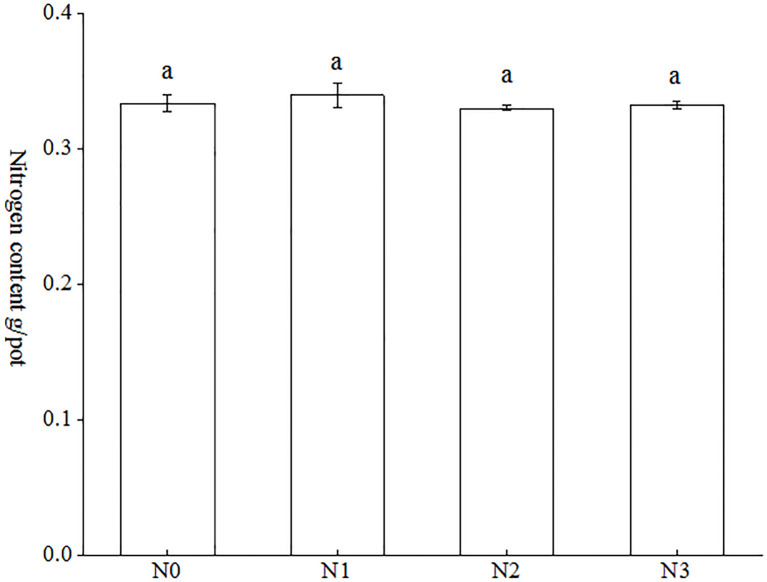
Effect of AMF on nitrogen accumulation in tobacco under different nitrogen forms. Different treatments are used with lower case letters (a, b, c) to indicate statistical significance (p<0.05).

As indicated in **Figure 4**, the nitrogen accumulation of tobacco plants in each treatment ranged from 0.3323 g/pot to 0.4099 g/pot. The nitrogen accumulation of each treatment exhibited the following hierarchy: N3 < N0 < N1 < N2. The data indicates that the N2 treatment exhibited the highest nitrogen accumulation, with a mean value of 0.4099 g/pot, while the N3 treatment demonstrated the lowest nitrogen accumulation, with a mean value of 0.3323 g/pot. The observed increase between the two treatments was 23.35%. Nevertheless, no statistically significant differences were observed between the various treatments.

### 3.4 Differences in the uptake of different forms of nitrogen by tobacco inoculated with AMF

**Figure 5:**
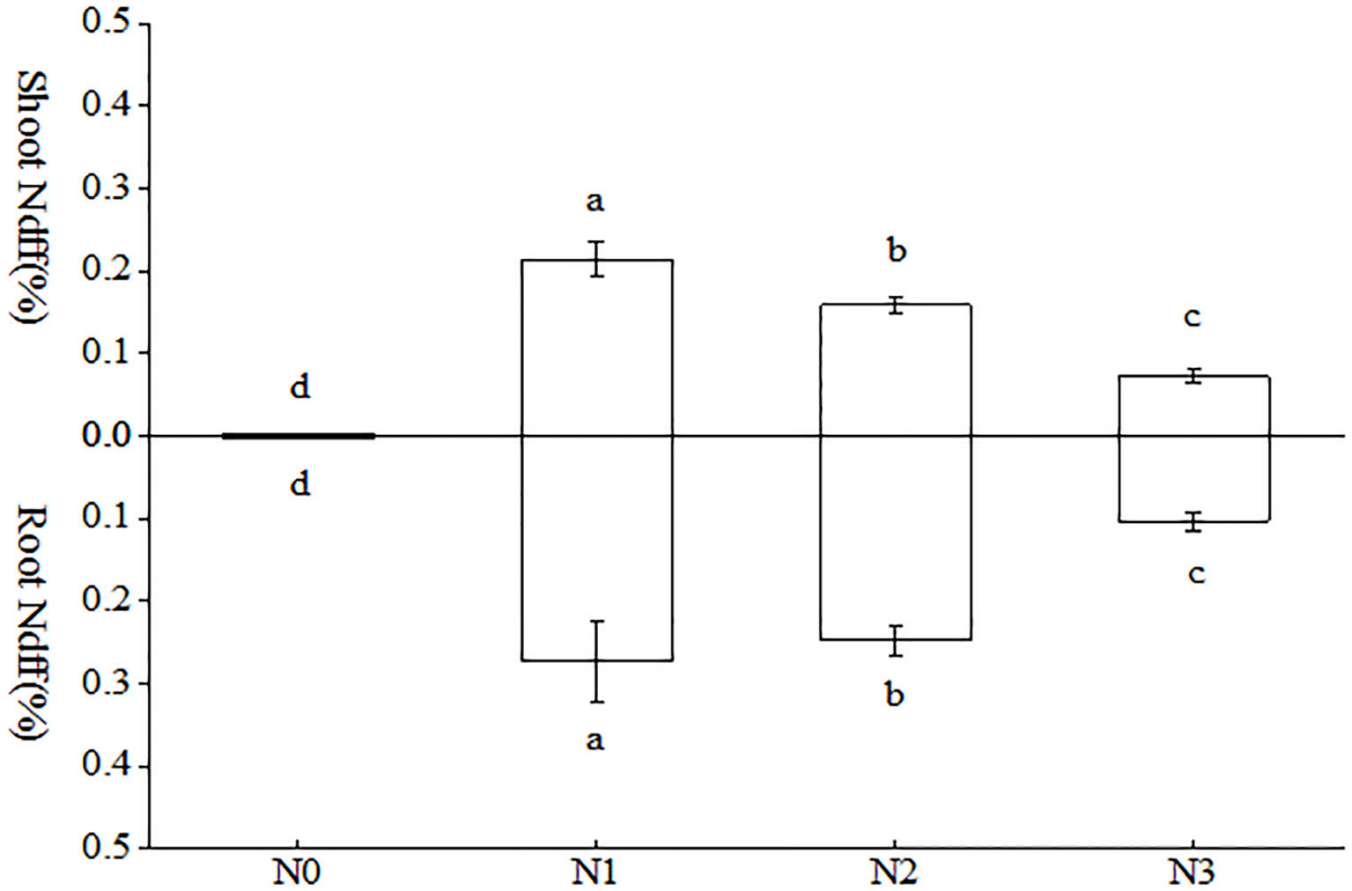
Differences of AMF uptake of different forms of nitrogen in tobacco. Different treatments are used with lower case letters (a, b, c) to indicate statistical significance (p<0.05).

As evident from **Figure 5**, there were notable discrepancies in the aboveground and belowground Ndff% among the various treatments. In the aboveground portion of the tobacco plant, the N1 treatment exhibited significantly higher levels of nitrogen compared to the other treatments. Notably, the N1 treatment was 1.35 and 2.94 times higher than the N2 and N3 treatments, respectively. In the subterranean portion of the tobacco plant, the N1 treatment exhibited a higher concentration, followed by the N2 treatment, with notable distinctions between the two. Interestingly, both treatments demonstrated a considerable elevation in comparison to the remaining treatments. In terms of Ndff% of the whole tobacco plant, the N1 treatment exhibited the highest level and was significantly higher than that observed in the other treatments. Compared to N2 and N3, respectively, there was an increase of 20.12% and 176.01%.

### 3.5 Proportion of uptake of different forms of nitrogen in tobacco by inoculation with AMF

**Figure 6:**
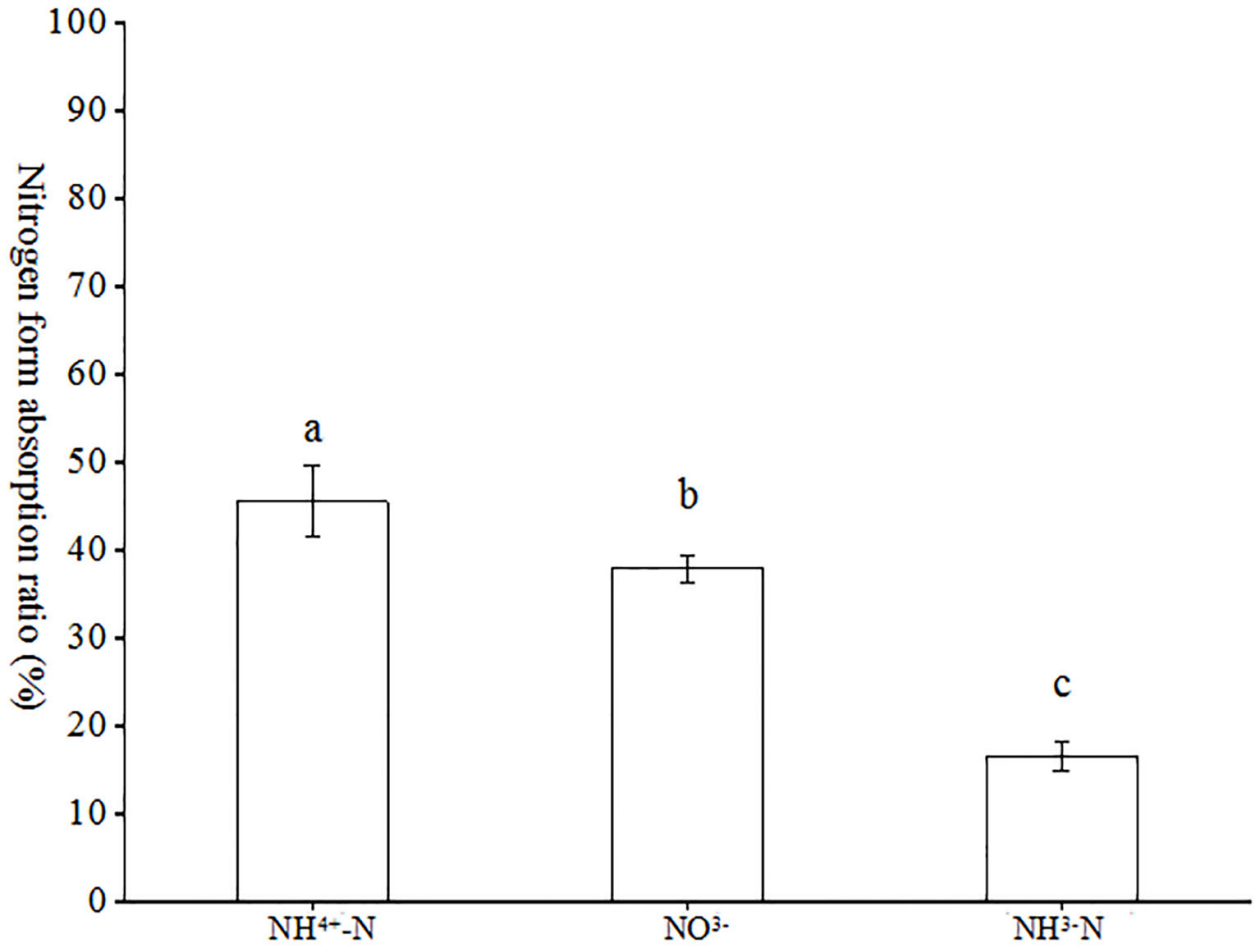
Absorption ratio of different forms of nitrogen in tobacco by AMF. Different treatments are used with lower case letters (a, b, c) to indicate statistical significance (p<0.05).

As illustrated in **Figure 6**, the ratios of each nitrogen form to the total nitrogen uptake in the tests with (NH_4_)_2_SO_4_, KNO_3_, Glu and their ^15^N markers exhibited notable differences, with (NH_4_)_2_SO_4_ exhibiting a significantly higher ratio than the remaining two forms, followed by nitrate nitrogen. The ratios of (NH_4_)_2_SO_4_, KNO_3_ and Glu in the total nitrogen forms were 48.61%, 36.10% and 15.29%, respectively. It can be observed that inorganic nitrogen constituted the primary nitrogen source absorbed by AMF. In the coexistence of ammonium, nitrate and glutamate nitrogen, AMF preferentially absorbed ammonium nitrogen, followed by nitrate nitrogen.

## 4 Discussion

The colonization rate of AMF serves as a critical parameter for evaluating their symbiotic effects on host plants, particularly in enhancing growth performance, nutrient acquisition efficiency, and stress tolerance capacity (Singh et al., 2024). Higher arbuscular mycorrhizal colonization rates are generally considered to indicate more significant improvements in plant N and P uptake, and stress tolerance (Xiang et al., 2023; Wu et al., 2024; Zeng et al., 2025). AMF colonization rate in tobacco varied with different strains, growth conditions and different tobacco varieties. For example, mycorrhizal colonization of tobacco seedlings cultured in soil and substrate inoculated with AMF alone was 77.1% and 20.13%, respectively (Liu et al., 2020; Zeng et al., 2025). Mycorrhizal colonization of tobacco reached 90.63% when inoculated only with *Glomus versiforme* in pot experiments (Begum et al., 2021b). A recent study indicated that mycorrhizal colonization of tobacco K326 cultured in soil was 26.43%, 48.30%, 92.22%, 100% and 81.11% when inoculated singly with *A. bireticulata*, *C. etunicatum*, *S. viscosum*, *F. mosseae* and *R. intraradices*, respectively (Yang et al., 2025). In the present study, the percentage of mycorrhizal colonization rate of tobacco cultured in autoclaved soil inoculated with *Claroideoglomus etunicatum* ranged from 31.13% to 35.19% (**Figure 2**). This may be attributed to the fact that the soil in this study was autoclaved as well as the different tobacco varieties planted. Moreover, there was no significant difference in tobacco bioaccumulation (**Figure 3**), and total nitrogen accumulation (**Figure 4**) between the different treatments in this study, which was presumed to be the same amount of nitrogen applied to the different treatments because the main objective of this study was to investigate the differences in uptake of different forms of nitrogen by tobacco, so the control conditions were set.

The symbiotic relationship between AMF and plant roots has globally important effects on nutrient. The classical view is that the contribution benefit of AMF symbiosis to plant nutrient acquisition is mainly related to P cycling, including plant P acquisition, P use efficiency, and reducing P losses from the plant-root system (Qiu et al., 2022; Li et al., 2024; Wu et al., 2024; Naseer et al., 2024). Emerging evidence indicates that AMF symbiosis plays a substantial role in enhancing plant nitrogen acquisition, potentially contributing up to 40% of total plant nitrogen uptake and reducing N losses in agroecosystems (Rui et al., 2022; Gojon, 2022; Howard et al., 2022; Zhang et al., 2023, 2024; Basiru et al., 2025). Recent advances have shed some light on the molecular mechanisms underlying AM-mediated nutrient acquisition, with the identification of fungal and plant genes critical for soil nutrient assimilation and symbiotic interfacial transport (Xie et al., 2022; Deng et al., 2022; Cheng et al., 2022; Bai et al., 2024; Basiru et al., 2025). The identification of nitrogen transporter proteins - including AMTs (NH_4_^+^), NRT2/NRT1 (NO_3_^−^), and LHT (amino acids) - that exhibit preferential or exclusive expression in AM-colonized root cells has advanced mechanistic understanding of AM-facilitated plant nitrogen acquisition (Gojon, 2022; Cheng et al., 2022; Wang et al., 2022; Han et al., 2023; Li et al., 2024). According to current knowledge, AMF absorbed mainly nitrate and ammonium through intra- and extra-root mycelium (Basiru et al., 2025). In terms of energy consumption, NH_4_^+^ is preferentially assimilated by AMF via the GS/GOGAT cycle compared to NO_3_^-^ (Rui et al., 2022). The results of this study confirmed that the ^15^N abundance in the N1 whole plant treatment was found to be 1.35 and 2.94 times higher than that observed in the N2 and N3 treatments (**Figure 5**), respectively. With regard to the total quantity of nitrogen absorbed, (NH_4_)_2_SO_4_, KNO_3_ and Glu were responsible for 48.61%, 36.10%, and 15.29%, respectively. The experimental results demonstrated that AMF-colonized tobacco plants exhibited significantly enhanced NH_4_^+^ assimilation efficiency compared to other nitrogen sources under mixed nitrogen conditions. Furthermore, several fungal AMT genes (*GintAMT1*, *GintAMT2*, GintAMT3, *LjAMT2*, and *LbAMT3-1*) encoding ammonia transporter have been identified within the symbiotic pathway between AMF and plants, thereby confirming the hypothesis that mycorrhizalized plants are more susceptible to ammonium ion uptake (Rui et al., 2022; Cheng et al., 2022; Wang et al., 2022). More interestingly, this study found that the Glu ^15^N-labeling treatment significantly exceeded the non-labeling treatment (N0), indicating that AMF can absorb a certain amount of Glu, although the absorption is relatively small. This phenomenon has been demonstrated that AM fungi can also absorb certain amounts of amino acids such as glycine, glutamic acid, Glu and aspartic acid (Rafael B S Valadares et al., 2021; Rui et al., 2022; Wu et al., 2024). The LjLHT1.2 gene encoding a LHT1-type amino acid transporter protein was identified to be expressed in AM root cortical cells, which further confirms that AMF can indeed take up certain amounts of amino acids (Rui et al., 2022). Integrated analyses demonstrated that AMF enhanced plant-mediated decomposition and acquisition of complex organic nitrogen sources in soil. These findings supported the deciphering of the mechanism of AM-mediated nitrogen nutrient uptake, and further validated the applicability of AMF in agricultural sustainability.

## 5 Conclusion

The primary nitrogen forms absorbed by AMF were investigated through experimentation using a *Claroideoglomus etunicatum* and Chinese Tobacco 100 (ZY100) as test materials, with (NH_4_)_2_SO_4_, KNO_3_, Glu and their corresponding ^15^N markers serving as the nitrogen sources. The results of this study showed that there was no significant difference in mycorrhizal colonization rate, biomass accumulation and nitrogen accumulation in tobacco by AMF in different treatments. In this pot-based study, soil sterilization by autoclaving to eliminate native AMF populations resulted in restricted mycorrhizal colonization rates of 31.13% - 35.29%. However, the findings revealed notable discrepancies in the uptake of distinct nitrogen forms by AMF. The uptake was markedly higher in the ^15^N-Glu-labelled treatment (N3) than in the unlabeled treatment (N0), indicating that AMF is capable of absorbing a certain quantity of Glu. In the case of the coexistence of (NH4)_2_SO_4_, KNO_3_ and Glu, AMF exhibited a preferential absorption of NH_4_^+^, which was 1.35 and 2.94 times higher than NO_3_- and Glu, respectively. The order of AMF uptake was found to be ^15^NH_4_^+^ > ^15^NO_3_^-^ > ^15^N-Gln. With regard to the total quantity of nitrogen absorbed, (NH_4_)_2_SO_4_, KNO_3_ and Glu were responsible for 48.61%, 36.10%, and 15.29%, respectively. These findings reinforce the understanding of the mechanisms of N nutrient acquisition in AMF symbiosis. To further verify the role of AMF in agricultural sustainability, evaluating N uptake preferences under field conditions or different AMF species is needed.

## Data Availability

The original contributions presented in the study are included in the article/supplementary material. Further inquiries can be directed to the corresponding author/s.
